# Adaptation and validation of the Online-Fear of Missing Out Inventory into Turkish and the association with social media addiction, smartphone addiction, and life satisfaction

**DOI:** 10.1186/s40359-022-00856-y

**Published:** 2022-06-18

**Authors:** Fuad Bakioğlu, Metin Deniz, Mark D. Griffiths, Amir H. Pakpour

**Affiliations:** 1 Department of Guidance and Psychological Counseling, Faculty of Education, Karamanoğlu Mehmetbey University, Karaman, Turkey; 2 Department of Guidance and Psychological Counseling, Faculty of Education, Bartın University, Bartın, Turkey; 3 International Gaming Research Unit, Psychology Department, Nottingham Trent University, 50 Shakespeare Street, Nottingham, NG1 4FQ UK; 4 Department of Nursing, School of Health and Welfare, Jönköping University, Gjuterigatan 5, 553 18 Jönköping, Sweden

**Keywords:** Fear of missing out (FoMO), Social media addiction, Smartphone addiction, Life satisfaction

## Abstract

**Background:**

In online environments, fear of missing out (FoMO) is where individuals become constantly preoccupied with what others are doing online and feel unable to log off in case they miss something. FoMO is a concept associated with the use of online social media (OSM; e.g., *Facebook* use, *Instagram* use) and various scales have been developed to assess the concept. One such scale is the Online Fear of Missing Out (On-FoMO) Inventory. The present study translated the On-FoMO Inventory into Turkish and its main aim was to test the validity and reliability of the scale. The secondary aim was to investigate the relationships between FoMO, social media addiction, smartphone addiction, and life satisfaction.

**Methods:**

A total of 419 participants (289 females and 130 males, mean age = 25.43 years, SD = 6.37) completed a self-report questionnaire including the On-FoMO Inventory, Fear of Missing Out Scale, Bergen Social Media Addiction Scale, Smartphone Addiction Scale-Short Version, and Satisfaction with Life Scale. In the adaptation process of the On-FoMO Inventory, confirmatory factor analysis, concurrent validity, and reliability analyses were performed.

**Results:**

The four-factor structure of the On-FoMO Inventory was confirmed and the Turkish version of the scale demonstrated good reliability. Online FoMO was positively related to social media addiction and smartphone addiction, and negatively related to life satisfaction.

**Conclusion:**

The results showed that the Turkish version of the On-FoMO Inventory has strong psychometric properties.

## Introduction

Over the past decade, the use of smartphones and social media has become more widespread worldwide. According to the Global Statshot Report 2021, there were 5.27 billion mobile phone users and 4.48 billion active social media users globally [1]. According to this report, Turkey (where the present study was carried out) ranks 44th among 238 countries in terms of the number of social media users in the general population. Moreover, the most frequently used social media platforms worldwide are *Facebook, YouTube, Instagram, Reddit, Snapchat,* and *Twitter* [2]. The concept of online social media (OSM) use discussed in the present study refers to the use of applications such as *Facebook, Instagram,* and *Twitter*. The usage rate of these applications appears to increase daily [2–4]. OSM use offers the opportunity for individuals to share information, documents, photographs, video, and audio in virtual environments. However, excessive use of OSM can lead to addiction in a minority of cases, disrupting the lives of such individuals. One of the possible contributory causes of OSM addiction is fear of missing out (FoMO), where individuals become constantly preoccupied with what others are doing online and feel unable to log off in case they miss something [5].

FoMO has been one of the most studied concepts in the past decade. FoMO has been defined as “a pervasive apprehension that others might be having rewarding experiences from which one is absent” [5]. In modern society, young people spend a lot of time posting things on social media, following current trends with their friends, and constantly updating their status [6, 7]. It has been reported that increased use of social media can lead to anxiety among some users with regards to missing out on new experiences and opportunities [8]. Individuals who experience FoMO have a desire to know what others are currently doing elsewhere [5]. When individuals have more opportunities or options for activities to participate in, they have more difficulty in choosing between them. Individuals who have difficulty in choosing an activity to attend may experience anxiety. When individuals choose a specific activity to participate in, the thought that they might have enjoyed another activity may also cause anxiety and lead to FoMO among some individuals [5, 9]. Today, social media applications have an important place in many individuals’ lives because they allow users to stay in touch with others, socializing, and communicating [10].

In the past decade, studies conducted with OSM users have reported a positive significant relationship between FoMO and social media addiction [11–14] and smartphone addiction [15–17]. Moreover, positive correlations have been found between FoMO and social media fatigue [18, 19], social media stalking, social comparison [18, 20], gaming disorder [21, 22], and impulsivity [21]. On the other hand, a significant negative correlation has been reported between FoMo and life satisfaction [23–25]. Since the use of OSM meets the needs of the individuals such as socializing and becoming popular, they may choose to stay in touch with others so that they do not miss out on social media updates [26].

Currently, there are only a few measures that assess FoMO among individuals. For example, Riordan et al. [4] developed a single-item FoMO measure (e.g., *“Do you experience FoMO?”*). The FoMO Scale was also developed to assess FoMO levels among individuals [5]. This scale is a uni-dimensional self-report scale and has been used in many studies [16], but only one item in the scale is relates specifically to online behavior (i.e., *“When I have a good time it is important for me to share the details online”*) [5]. Since FoMO appears to be related to OSM use, the Online Fear of Missing Out (On-FoMO) Inventory was developed [27]. Moreover, research indicates that FoMO is related to internet addiction and OSM use [28–30].

The On-FoMO Inventory consists of four dimensions (need to belong, need for popularity, anxiety, and addiction) [27]. More specifically, in relation to FoMO, (i) the need to belong refers to being a part of the group [5, 31]; (ii) the need for popularity refers to seeking approval from others and having high self-esteem [4, 27], (iii) anxiety refers to the emotional problems faced in situations where access to OSM is blocked or impossible [27, 32, 33]; and (iv) addiction refers to OSM use at a level that prevents individuals from engaging in daily activities (e.g., sleeping, eating, fulfilling educational and occupational-related responsibilities) [7, 27, 34, 35].

OSM use appears to be increasing daily in Turkey as well as in other countries all around the world [1]. Therefore, the use of the four-factor On-FoMO Inventory in studies on FoMO in Turkey will ensure that FoMO is addressed across all its dimensions. Based on the aforementioned literature, it appears that the On-FoMO Inventory is appropriate for research on FoMO in Turkey. However, the scale has only been validated in English. Therefore, the main purpose of the present study was to translate and validate the On-FoMO Inventory into Turkish. Individuals experience FoMO while constantly following others online. Individuals with FoMO may experience addiction because they constantly use social media and do not allow themselves to be without their smartphones. All these factors can reduce the life satisfaction of some individuals. A secondary aim of the present study was to examine the relationships between FoMO, social media addiction, smartphone addiction, and life satisfaction.

## Methods

### Participants and procedure, and ethics

The present study’s sample comprised 419 volunteer participants recruited by convenience sampling [69.0% females (n = 289) and 31.0% males (n = 130)]. The ages of the participants ranged from 18 to 38 years (M = 25.43 SD = 6.37). The descriptive information of the participants is given in Table 1. A cross-sectional online survey (hosted on *Google Forms*) was used to collect the data between May 2021 and June 2021. Online platforms (*Facebook, Instagram, Twitter, and WhatsApp*) were used to recruit the participants. Answering the survey took approximately 15–20 min. Some eligibility criteria were considered for participation in the present study. Participants had to (i) have one or more social media accounts; (ii) be aged 18 years or older; and (iii) provide informed consent. While answering the survey, the anonymity and confidentiality of the participants were taken into consideration. The present study was conducted in accordance with the Declaration of Helsinki and was approved by the ethics committee of the first author’s university.

**Table 1:** Descriptive information of the participants (n = 419)

	Frequency	%
*Gender*		
Female	289	69
Male	130	31
*Number of social media accounts*		
1–2	179	42.7
3–4	178	42.5
5 or more	62	14.8
*Time spent checking call notifications during the day*		
0–1 h	280	66.8
1–2 h	63	15.0
Above 2 h	65	15.5
No checking	11	2.6
*Time spent checking Facebook notifications during the day*		
0–1 h	143	34.1
1–2 h	36	8.6
Above 2 h	90	21.5
No checking	69	16.5
No Facebook account	81	19.3
*Time spent checking Twitter notifications during the day*		
0–1 h	135	32.2
1–2 h	45	10.7
Above 2 h	95	22.7
No checking	48	11.5
No Twitter account	96	22.9
*Time spent checking Instagram notifications during the day*		
0–1 h	197	47.0
1–2 h	100	23.9
Above 2 h	88	21.0
No checking	15	3.6
No Instagram account	19	4.5
*Time spent checking e-mail notifications during the day*		
0–1 h	160	38.2
1–2 h	47	11.2
Above 2 h	156	37.2
No checking	56	13.4
*Time spent checking YouTube notifications during the day*		
0–1 h	129	30.8
1–2 h	82	19.6
Above 2 h	179	42.7
No checking	29	7.0
*Time spent checking WhatsApp notifications during the day*		
0–1 h	268	64.0
1–2 h	52	12.4
Above 2 h	94	22.4
No checking	5	1.2

### Translation process

Turkish adaptation of the online FoMO scale was performed using a standard procedure [36]. First, two native Turkish translators translated the On-FoMO Inventory from English to Turkish. Both interpreters were well versed in both psychology and the scope of the scale. Second, two experts in psychology integrated the two Turkish translations into one form. Third, two experts in psychology back-translated the scale from Turkish to English. Finally, two psychology experts compared the original English form of the scale, the Turkish translation, and the Turkish-English translation, and decided on the final Turkish form of the scale.

### Measures

#### Online Fear of Missing Out Inventory (On-FoMO)

The 20-item On-FoMO [27] was used to assess fear of missing out online. The scale comprises four factors (i.e., need to belong, need for popularity, anxiety, and addiction) and items (e.g., *Need to belong*; I feel distant from people when I see them happy in posts. *Need for popularity*; I need people to like or comment on my posts. *Anxiety*; I get anxious when my cell phone does not have internet signal. *Addiction*; When I’m on social networks, I forget my problems) are rated on a four-point scale from 1 (*has nothing to do with me*) to 4 (*has a lot to do with me*). The scores range from 20 to 80 and higher scores indicate greater fear of missing out online. The Cronbach’s alpha internal consistency coefficients were good in the original study (total scale α = 0.92, need to belong α = 0.84, need for popularity α = 0.81, anxiety α = 0.85, and addiction α = 0.73). In this study, the Cronbach’s alpha internal consistency of the scale were good (scale total α = 0.93, need to belong α = 0.80, need for popularity α = 0.82, anxiety α = 0.89 and addiction α = 0.81).

#### Fear of Missing Out Scale (FoMOS)

The 10-item unidimensional FoMOS [5, 7] was used to assess fear of missing out. Items (e.g., I feel that I have a number of good qualities) are rated on a five-point scale from 1 (*not at all true of me*) to 5 (*extremely true of me*). The scores range from 10 to 50 and higher scores indicate greater fear of missing out. The Cronbach's alpha internal consistency estimates were good in the original studies (α = 0.87-0.90). In the present study, the Cronbach’s alpha internal consistency coefficient of the scale was good (α = 0.81).

#### Bergen Social Media Addiction Scale (BSMAS)

The six-item unidimensional BSMAS [37, 38] was used to assess the risk of social media addiction. Items (e.g., How often during the last year have you felt an urge to use social media more and more?) are rated on a five-point scale from 1 (*very rarely*) to 5 (*very often*). The scores range from 6 to 30 and higher scores indicate greater risk of social media addiction. The Cronbach’s alpha internal consistency of the scale was good in the original study (α = 0.88). In the present study, the Cronbach’s alpha internal consistency coefficient of the scale was good (α = 0.86).

#### Smartphone Addiction Scale-Short Version (SAS-SV)

The 10-item unidimensional SAS-SV [39, 40] was used to assess the risk of smartphone addiction. Items (e.g., Using my smartphone longer than I had intended) are rated on a six-point scale from 1 (*strongly disagree*) to 6 (*strongly agree*). The scores range from 10 to 60 and higher scores indicate greater risk of smartphone addiction. The Cronbach’s alpha internal consistency of the scale was excellent in the original study (α = 0.91). In the present study, the Cronbach’s alpha internal consistency coefficient of the scale was excellent (α = 0.92).

#### Satisfaction With Life Scale (SWLS)

The five-item unidimensional SWLS [41, 42] was used to assess life satisfaction. Items (e.g., The conditions of my life are excellent) are rated on a five-point scale from 1 (*totally disagree*) to 5 (*totally agree*). The scores range from 5 to 25 and higher scores indicate greater life satisfaction. The Cronbach’s alpha internal consistency of the scale was good (α = 0.87) in the original study. In the present study, the Cronbach’s alpha internal consistency coefficient of the scale was good (α = 0.81).

#### Demographic variables

Participants were asked about their age, gender, the number of social media accounts, and how often to calls during the day. Participants were also asked how often they checked social media platforms (*Facebook, Twitter, Instagram, e-mail, YouTube,* and *WhatsApp*) during the day.

### Data analysis

Reliability of the On-FoMO Inventory was assessed using internal consistency, average variance extracted (AVE) and composite reliability (CR). To assess factor structure of the On-FoMO Inventory, confirmatory factor analysis (CFA) was used. More specifically, the diagonally weighted least squares (DWLS) estimator was used in the CFA due to the ordinal nature of the data. Several model fit indices were used to test CFA model: root mean square approximation error; comparative fit index; Tucker-Lewis index; and standardized root mean square residual. Acceptable fit values are RMSEA and SRMR ≤ 0.08, CFI, NFI and TLI ≥ 0.90 [43, 44]. Furthermore, descriptive statistics, item factor loadings, and corrected item-total correlation coefficients for each item in the scale were examined.

Rasch analysis was used to test structure of the On-FoMO Inventory. A partial credit model was applied to scale the data. Item fit was assessed using information-weighted fit statistic (infit), mean square (MnSq), and outlier-sensitive fit statistic (outfit) MnSq. An item with acceptable fit has MnSq vales between 0.5 and 1.5 [45]. Measurement invariance across gender was tested using the differential item functioning (DIF). A DIF contrast > 0.5 indicates substantial variance across gender groups.

Network analysis was also conducted to test further On-FoMO Inventory structure. A network has two components: node (observed variables or items) and edge (the magnitude of relationships between the observed variables or nodes). The network analysis was conducted using EBICglasso estimator (Extended Bayesian Information Criterion Graphical Least Absolute Shrinkage and Selection Operator) to estimate the network. Three centrality indices were calculated including betweenness, closeness, and strength. JASP version 0.15.0 and R packages bootnet, networkTools, and qgraph were used to estimate network analysis.

The relationships between online FoMO, social media addiction, smartphone addiction and life satisfaction were examined to ensure the concurrent validity of the scale. Using PROCESS macro version 4.0 with Bootstrapping method analysis was performed to determine the significance of direct and indirect effects in larger samples. For the bootstrapping analysis, 10,000 resamples and a 95% confidence interval were determined. IBM SPSS Statistics 23.0, Amos Graphics 23 (IBM Crop. Armonk, NY), WINSTEPS software version 5.1.1 and R software with the lavaan package were used for the analysis in the present study.

## Results

### Confirmatory factor analysis

As a result of CFA performed for the Turkish version of the On-FoMO Inventory, all goodness-of-fit indices were within the acceptance range (χ^2^ = 10,608.620, df = 190, p = 0.051; CFI = 0.997, NNFI = 0.997, TLI = 0.997, RMSEA = 0.021, SRMR = 0.054). The factor load values of the scale were found to be between 0.43 and 0.97, and the corrected item-total correlations were found to be between 0.43 and 0.73 (see Table 2).

**Table 2:** Psychometric properties of the On-FoMO Inventory at the item level

Item^#^	Factor loading*^†^	Item−total correlation	Mean (SD)	S	K		Infit MnSq	Outfit MnSq	Difficulty	Discrimination	DIF contrast across gender ^§^^,^^¶^
Item 1	0.700	.53	2.72 (1.03)	−.21	−1.13		1.24	1.35	−0.98	0.71	0.31
Item 2	0.732	.61	2.03 (1.04)	.66	−.79		0.89	0.90	−0.58	1.01	−0.32
Item 3	0.783	.57	2.66 (1.06)	−.21	−1.18		0.97	0.92	−0.79	1.06	−0.34
Item 4	0.969	.73	2.17 (1.07)	.42	−1.11		1.03	0.97	0.82	1.01	−0.45
Item 5	0.925	.68	2.26 (1.05)	.33	−1.11		0.69	0.68	0.53	1.38	0.35
Item 6	0.531	.52	1.58 (.92)	1.45	.96		1.24	1.17	0.48	0.88	0.15
Item 7	0.743	.58	2.07 (1.12)	.60	−1.06		1.03	0.98	−0.67	0.97	0.15
Item 8	0.832	.66	2.28 (1.01)	.29	−.99		1.07	1.10	0.43	0.88	0.10
Item 9	0.797	.72	1.68 (.93)	1.18	.31		0.69	0.69	0.13	1.37	00
Item 10	0.558	.48	1.89 (1.01)	.76	−.66		1.50	1.50	−0.41	0.67	−0.15
Item 11	0.706	.68	1.54 (.87)	1.59	1.57		0.75	0.69	0.54	1.35	0.18
Item 12	0.429	.43	1.55 (.84)	1.44	1.13		1.28	1.09	0.53	0.80	00
Item 13	0.856	.64	2.04 (1.11)	.58	−1.08		0.80	0.82	−0.78	1.27	00
Item 14	0.751	.67	1.74 (.99)	1.06	−.15		0.86	0.81	0.06	1.20	−0.32
Item 15	0.652	.54	1.97 (1.02)	.68	−.74		1.33	1.43	−0.11	0.68	0.26
Item 16	0.523	.56	1.50 (.85)	1.67	1.86		1.17	1.12	0.71	0.93	0.46
Item 17	0.696	.56	2.05 (1.05)	.63	−.84		1.13	1.15	−0.29	0.84	−0.04
Item 18	0.757	.63	2.09 (1.02)	.55	−.85		0.69	0.71	−0.38	1.36	−0.36
Item 19	0.569	.60	1.77 (.82)	.97	.52		0.89	0.86	0.39	1.04	0.46
Item 20	0.699	.58	1.77 (1.03)	1.21	.22		0.96	0.80	0.39	1.31	−0.39

MnSq = mean square error; DIF = differential item functioning; S = Skewness; K = Kurtosis

*All factor loadings were significant at *p* < 0.001

^†^Based on the first-order confirmatory factor analysis (CFA)

^§^DIF contrast > 0.5 indicates substantial DIF

^#^DIF contrast across gender = Difficulty for male-Difficulty for female

### Reliability analysis

The results of the Rasch analysis are presented in Table 3. The most difficult item was Item 4 while the easiest item was Item 1. All items fitted satisfactorily in their own subcontracts (infit MnSq = 0.69 to 1.50; outfit MnSq = 0.69 to 1.50). No DIF item was detected for all items (DIF contrast = −0.45 to 0.46). The test–retest reliability of the On-FoMO Inventory was determined by administering the same scale to 85 participants with two-week interval. All test–retest reliability coefficients of the Inventory were good (Inventory total *r* = 0.84, need to belong *r* = 0.78, need for popularity *r* = 0.78, anxiety *r* = 0.89 and addiction *r* = 0.84).

**Table 3:** Psychometric properties of the at the scale level

Psychometric testing	Anxiety	Need to belong	Need for popularity	Addiction
Composite reliability	0.93	0.79	0.81	0.81
Average variance extracted	0.72	0.44	0.47	0.46
Internal consistency (Cronbach’s α)	0.902	0.796	0.821	0.814
McDonald’s ω	0.903	0.802	0.832	0.817
Item separation reliability from Rasch	0.98	0.98	0.97	0.94
Item separation index from Rasch	7.93	6.73	5.86	4.04
Person separation reliability from Rasch	0.84	0.76	0.77	0.76
Person separation index from Rasch	2.29	2.13	2.15	0.214

**Fig. 1:**
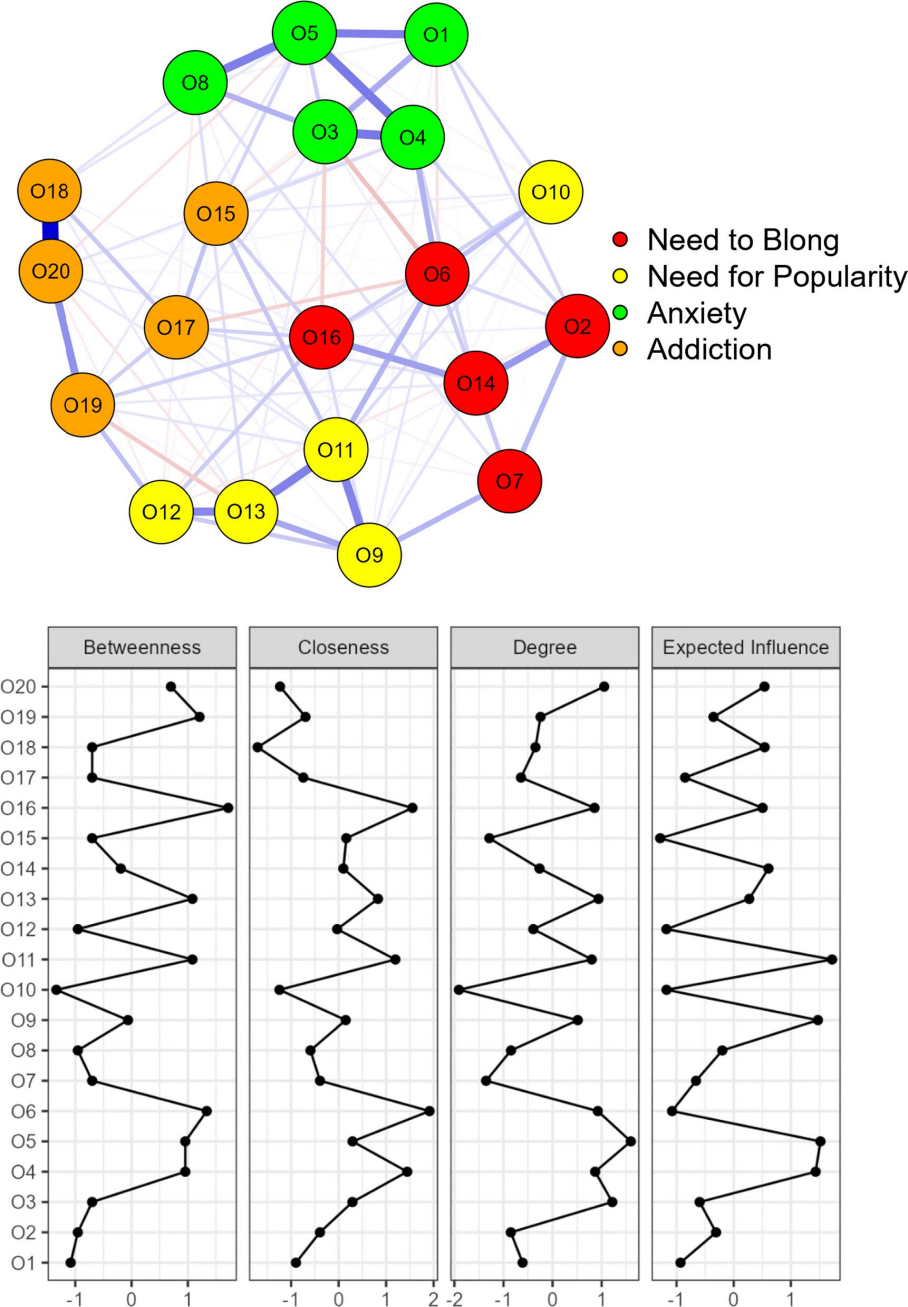
The network structure of On-FoMO Inventory domains. The edges represent regularized partial correlations. Blue lines show positive associations. Red lines would have indicated negative ones

The results of the network analysis are shown in Fig. 1. The network structure confirmed that On-FoMO Inventory had four distinct subscales. Of 190 possible edges, 113 (59.5%) were present with a sparsity value of 0.40. Item 18 and Item 20 showed the strongest correlations (r = 0.686), followed by Item 4 and Item 5 (r = 0.358).

### Concurrent validity analysis

For the concurrent validity analysis of the scale, Pearson correlation coefficient values were calculated between the On-FoMO Inventory and FoMO, social media addiction, smartphone addiction, and life satisfaction scales. Data analysis showed significant positive correlations between the On-FoMO Inventory and FoMO (*r* = 0.70), social media addiction (*r* = 0.67), and smartphone addiction (*r* = 0.78). On the other hand, the On-FoMO Inventory was significantly and negatively correlated with life satisfaction (*r* = −0.32). As Table 4 indicates, the On-FoMO Inventory significatory correlated with social media addiction, smartphone addiction, and life satisfaction (all *p*-values < 0.001).

**Table 4:** Correlation matrix of variables

Measure	1	2	3	4	5
1. Online FoMO	–				
2. FoMO	.697^**^	–			
3. Social media addiction	.671^**^	.529^**^	–		
4. Smartphone addiction	.781^**^	.583^**^	.768^**^	–	
5. Life satisfaction	−.324^**^	−.288^**^	−.295^**^	−.304^**^	–

^**^Correlation is significant at the *p* < 0.001 level (2-tailed)

**Fig. 2:**
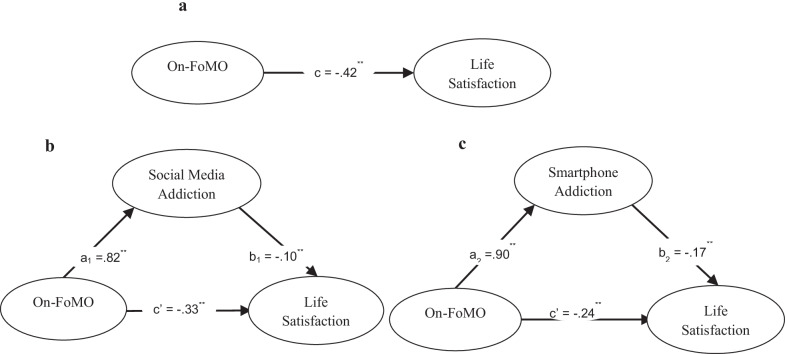
Mediated outcomes on life satisfaction showing indirect effects of On-FOMO through social media addiction and smartphone addiction

The present study investigated the relationships between online FoMO, social media addiction, smartphone addiction and life satisfaction (see Fig. 2). The findings of the research model are presented in Table 5. The direct effects of online FoMO on mediations (social media addiction: *B* = 0.319; LLCI = 0.285; ULCI = 0.353, smartphone addiction: *B* = 0.731; LLCI = 0.674; ULCI = 0.787) and life satisfaction were significant (*B* = −0.063; LLCI = -0.109; ULCI = −0.017). As a result of bootstrapping analysis, social media addiction (*B* = −0.024; LLCI = −0.057; ULCI = −0.006) and smartphone addiction (*B* = −0.016; LLCI = −0.056; ULCI = −0.002) mediated the relationship between online FoMO and life satisfaction.

**Table 5:** Mediation effects concerning study variables

Variable	B	SE (Bootstrapping SE)	*t*-value (Bootstrapping LLCI)	*p*-value (Bootstrapping ULCI)
Total effect of On-FoMO on life satisfaction^a^	*−0.100*	0.014	−7.005	< 0.001
Direct effect of On-FoMO on life satisfaction	−0.063	0.023	−2.705	0.0071
Direct effect of On-FoMO on mediators				
Social media addiction	0.319	0.017	18.463	< 0.001
Smartphone addiction	0.731	0.029	25.551	< 0.001
Indirect effect of On-FoMO on life satisfaction				
Total indirect effect	−0.039	(0.010)	(−0.072)	(−0.002)
Social media addiction	−0.024	(0.009)	(−0.057)	(−0.006)
Smartphone addiction	−0.0156	0.007	(−0.056)	(−0.002)

Bootstrap sample size = 10,000

SWLS, life satisfaction; LL, lower limit; UP, upper limit

^a^Total effect

## Discussion

The present study adapted and validated the Turkish version of the On-FoMO Inventory [27] and investigated the relationships between online FoMO, social media addiction, smartphone addiction, and life satisfaction. As a result of the CFA, the validity of the On-FoMO Inventory, which comprised 20 items and four dimensions, was confirmed with the Turkish sample. Factor loading values of scale items ranged between 0.55 and 0.88. Adjusted item-total correlation values, on the other hand, ranged between 0.43 and 0.73. The values obtained in the Turkish version of the On-FoMO Inventory were found to be close to the values obtained in the original validation study [27]. Item factor loads of the scale ≥ 0.40 and above are considered sufficient [46, 47]. Based on these results, it can be stated that the Turkish version of the inventory confirmed the factor structure of the original scale.

Reliability of the On-FoMO Inventory was assessed using internal consistency, average variance extracted and composite reliability. Cronbach’s alpha value for the original study ranged from 0.73-0.92 for the whole scale and its sub-dimensions [27]. In the present study, Cronbach’s alpha was evaluated and the reliability coefficients for the whole scale and its sub-dimensions ranged from 0.80 to 0.93. The results of reliability tests of the scale also met the criterion of having a Cronbach’s alpha value of 0.70 or above [48].

As a result of the concurrent validity analysis, positive correlations were found between online FoMO and FoMO, social media addiction, and smartphone addiction, and a negative correlation between online FoMO and life satisfaction. In the structural equation model, it was found that social media addiction and smartphone addiction mediated the relationship between online FoMO and life satisfaction. These results indicate that social media addiction and smartphone addiction increase and life satisfaction decreases as online FoMO level increases. As a result of the bootstrapping analysis, it was found that the significance of the results with larger samples did not change.

Studies have demonstrated that FoMO is positively related to social media addiction [12–14, 49] and smartphone addiction [16, 17]. For example, in a study conducted of Italian undergraduates, FoMO was positively associated with problematic internet addiction, and It has been found that there is a mediating role of positive metacognitions between FoMO and problematic social media addiction [12]. In another study conducted with university students, FoMO was positively associated with social network site use and smartphone addiction [16]. On the other hand, FoMO is negatively related with life satisfaction [23–25]. These findings indicate that as the individual’s FoMO level increased, social media addiction and smartphone addiction increased and life satisfaction decreased.

The present study has some limitations. First, data were collected from non-clinical participants. The results of the study cannot be generalized to individuals in clinical populations. Second, in the present study, test–retest reliability was not conducted. Third, the data collection tools used in the present study were self-report tools that carry the risk of response bias. Fourth, the present study was conducted during the COVID-19 pandemic, and an online data collection method was used instead of face-to-face method to minimize the risk of infection. Finally, the widespread use of online technologies in the COVID-19 pandemic period meant that individuals may have become more addicted to online activities than at other times.

## Conclusion

Despite the limitations, the findings show that the Online FoMO Inventory is a reliable and valid tool for determining the levels of online fear of missing out among Turkish samples. In the present study, social media addiction and smartphone addiction mediated the relationship between online FoMO and life satisfaction. These results indicate that social media addiction and smartphone addiction are important variables in the indirect effect of FoMO on life satisfaction. FoMO was associated with social media addiction and smartphone addiction [27]. FoMO can be both a contributory cause and the result of these addictions. Moreover, the On-FoMO Inventory has four factors (i.e., need to belong, need for popularity, anxiety, and addiction). As a result, the On-FoMO Inventory can be used by mental health professionals and researchers in studies investigating FoMO among the general population. The On-FoMO Inventory can be utilized by researchers as a useful assessment tool in identifying FoMO levels among Turkish individuals and testing the effectiveness of psychoeducational programs for individuals with high FoMO levels.

## Data Availability

The datasets used and/or analyzed during the current study are available from the corresponding author on reasonable request.
